# Characterization of 27 Mycotoxin Binders and the Relation with *in Vitro* Zearalenone Adsorption at a Single Concentration

**DOI:** 10.3390/toxins7010021

**Published:** 2015-01-05

**Authors:** Thomas De Mil, Mathias Devreese, Siegrid De Baere, Eric Van Ranst, Mia Eeckhout, Patrick De Backer, Siska Croubels

**Affiliations:** 1 Department of Pharmacology, Toxicology and Biochemistry, Faculty of Veterinary Medicine, Ghent University, Salisburylaan 133, 9820 Merelbeke, Belgium; E-Mails: mathias.devreese@ugent.be (M.D.); siegrid.debaere@ugent.be (S.D.B.); patrick.debacker@ugent.be (P.D.B.); siska.croubels@ugent.be (S.C.); 2 Department of Geology and Soil Science, Faculty of Science, Ghent University, Krijgslaan 281, S8, 9000 Ghent, Belgium; E-Mail: eric.vanranst@ugent.be; 3 Department of Applied Biosciences, Faculty of Bioscience Engineering, Ghent University, Valentin Vaerwyckweg 1, 9000 Ghent, Belgium; E-Mail: mia.eeckhout@ugent.be

**Keywords:** mycotoxin, binders, characterization, zearalenone, adsorption screening

## Abstract

The aim of this study was to characterize 27 feed additives marketed as mycotoxin binders and to screen them for their *in vitro* zearalenone (ZEN) adsorption. Firstly, 27 mycotoxin binders, commercially available in Belgium and The Netherlands, were selected and characterized. Characterization was comprised of X-ray diffraction (XRD) profiling of the mineral content and *d*-spacing, determination of the cation exchange capacity (CEC) and the exchangeable base cations, acidity, mineral fraction, relative humidity (RH) and swelling volume. Secondly, an *in vitro* screening experiment was performed to evaluate the adsorption of a single concentration of ZEN in a ZEN:binder ratio of 1:20,000. The free concentration of ZEN was measured after 4 h of incubation with each of the 27 mycotoxin binders at a pH of 2.5, 6.5 and 8.0. A significant correlation between the free concentration of ZEN and both the *d*-spacing and mineral fraction of the mycotoxin binders was seen at the three pH levels. A low free concentration of ZEN was demonstrated using binders containing mixed-layered smectites and binders containing humic acids.

## 1. Introduction

The contamination of feed with mycotoxins is a continuing feed safety issue, leading to economic losses in animal production [1]. Consequently, a variety of methods for the decontamination of feed has been developed, but the addition of mycotoxin detoxifiers to the feed is the most commonly-used method [2,3]. The additives used for this purpose can be divided into two groups: binders and modifiers. Mycotoxin binders aim to prevent the absorption of the mycotoxins from the intestinal tract of the animal by adsorbing the toxins to their surface. Mycotoxin binders are generally clay- (inorganic) or yeast-derived (organic) products [3]. Mycotoxin modifiers, on the other hand, aim to alter the chemical structure of the mycotoxins and, consequently, reduce their toxicity. Mycotoxin modifiers are usually of microbiological origin comprised of whole cultures of bacteria or yeasts, as well as specifically extracted components, such as enzymes [4].

The extensive use of specialized additives to diminish the effects of mycotoxins has led to the establishment of a new group of feed additives in 2009: “substances for reduction of the contamination of feed by mycotoxins: substances that can suppress or reduce the absorption, promote the excretion of mycotoxins or modify their mode of action” [5]. However, most of the mycotoxin detoxifiers are registered as technical additives, feedstuff or digestibility enhancers, as those are more easily being registered in comparison to the claim of a mycotoxin detoxifier. At the moment, only two products are registered as being a mycotoxin detoxifier [6], whereas a wide variety of products indirectly claiming mycotoxin binding or modifying abilities is available. In addition, European legislation does not require full transparency with regard to the content of these technical additives.

Although many different types of ingredients are known to be used in additives marketed as mycotoxin binders (in brief, binders), no studies are available that provide a comprehensive overview of their exact composition. In most reports, the description of the products is limited to the product name and an entry of a generic name, such as hydrated sodium calcium aluminosilicate (HSCAS) or bentonite [3]. Despite this generic nomenclature of commercially-available binders, several physicochemical properties have been identified as having a possible correlation with adsorption of mycotoxins and might therefore be used to categorize the different available products. These characteristics originate from soil science and comprise cation exchange capacity (CEC), exchangeable K^+^, Na^+^, Mg^++^ and Ca^++^, acidity, linear swelling, mineral fraction and relative humidity [7]. 

Exchangeable cations neutralize the interlayer charges in phyllosilicates and are involved in the binding mechanism of aflatoxin B1 [8,9]. The CEC is a measure of the amount of exchangeable cations, whereas the different types of exchangeable base cations (K^+^, Na^+^, Mg^++^ and Ca^++^) have different properties in terms of their affinity for the clay and osmolarity [10]. Although a correlation between the binding properties of mycotoxins and CEC values is not documented in the literature, this parameter is cited by manufacturers when discussing the binding properties of inorganic mycotoxin binders. 

The pH of the binder can provide insight into the saturation of a clay with exchangeable base cations, which results in a pH of seven or higher. An increase in pH can be due to the solvation of the exchangeable base cations or the presence of carbonates. A low pH is indicative for exchangeable Al^3+^ or the presence of acidic functional groups, e.g., humic acids. 

Adsorption to clays is not limited to the surface of the clay particles, but extends also to the interlayer space of the clay. This interlayer space, characterized by the *d*-spacing, can be determined with X-ray diffraction (XRD) and is restrictive for the formation of one or more adsorbent layers. This space can increase if the clay swells, thereby increasing the number of binding sites [11]. Hydration of the minerals plays an important role in this process, as well, since it is related to the osmotic power of the mineral [12,13] and, hence, the ability to hydrate the interlayer space. 

Non-enzymatic organic compounds used in feeds are mostly products derived from yeast cell walls or organic mineraloids, such as leonardite and lignite, which are a rich source of humic and fulvic acids. Adsorption to these compounds can occur through hydrophobic interactions [14]. Such interactions were proposed for the binding of the antibiotic, oxytetracycline, to montmorillonites in the presence of dissolved organic matter [15]. To determine the mineral fraction of a sample, the organic compounds are discarded by dry combustion. 

With regard to the adsorption of mycotoxins, zearalenone (ZEN) is a secondary metabolite produced by several fungi of the *Fusarium* genus. It has lipophilic properties and exerts its effects on the reproductive system of animals [16,17]. Sabater-Vilar *et al.* described the ZEN-adsorption of three smectite-based minerals, six humic substances, four yeast-derived detoxifiers and six commercial products, which include, according to the commercial brochures, two yeast products, three mineral binders and a mixture of clay and yeast products. A large variation in the adsorption of ZEN is seen in all of the types of binders [18]. Yiannikouris *et al.* compared the ZEN binding properties of a yeast cell extract and a mineral binder and concluded that the yeast-based product had better adsorption properties than the mineral in the higher concentration range [19]. Avantaggiato *et al.* studied 19 binders and also found a large variation in ZEN adsorption [20]. These results indicate that ZEN can be adsorbed, but only by a limited number of binders, and there is a large variation in binding percentage. Therefore, ZEN binding can be used as model to evaluate which physicochemical properties are related to the binding of rather lipophilic mycotoxins. All of the studies cited above used activated carbon or charcoal as the positive control and found binding percentages of over 90%.

The first aim of this study was to identify the qualitative composition of 27 commercially-available feed additives marketed as mycotoxin binders by XRD analysis and to determine the following physicochemical properties: CEC, exchangeable K^+^, Na^+^, Mg^++^ and Ca^++^, acidity, swelling, mineral fraction, presence of carbonates (HCl effervescence test) and relative humidity.

The second aim was to discuss the relation between the observed free concentration of ZEN after incubation with the mycotoxin binders and the physicochemical properties of these binders. 

## 2. Results and Discussion

### 2.1. Physicochemical Characterization

The physicochemical properties of the 27 binders are presented in Table 1. These samples represent the vast majority of additives marketed as mycotoxin binders in Belgium and The Netherlands and are available in most European countries. All binders contain one or more mineral constituent, and some products contain organic compounds. Most binders are mixtures of different mineral constituents, and most prevalent compounds are smectites, such as montmorillonite. The ratio of exchangeable base cations varies widely, even among products with similar compounds. The non-mineral content of a binder with a low mineral fraction (*i.e.*, Sample Numbers 5, 12, 15 and 16) was confirmed by information provided by the manufacturer of the binder, who labelled these products as containing humic acids, leonardite or yeast-derived binders. 

### 2.2. Zearalenone Adsorption Screening and Correlation with Physicochemical Characteristics

**Figure 1:**
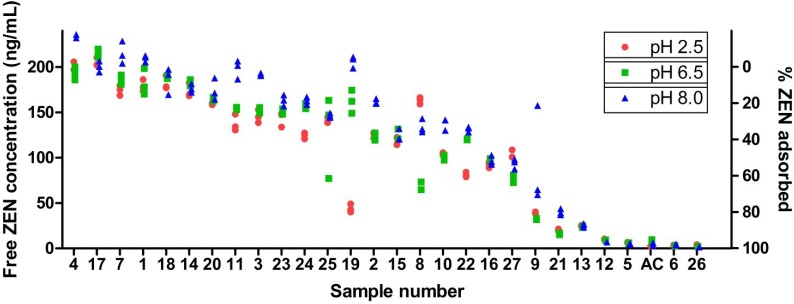
Free zearalenone (ZEN) concentration after incubation of ZEN with 27 mycotoxin binders at three different pHs (Sample Numbers 1–27). Individual results of three replicates are shown. AC represents activated carbon, which is included as the positive control.

The *in vitro* ZEN adsorption is assessed using a high throughput screening model applied at different pHs, which are representative for the gastro-intestinal tract of most monogastric animals. Similar models were successfully applied in previous *in vitro* experiments [18,19,20,21,22,23,24]. Major differences include the use of other buffer systems or media and the construction of adsorption isotherms. The use of other buffers or media may influence chemical equilibria, whereas adsorption isotherms may reveal information on the binding mechanism, affinity and capacity. This study focused on the determination of the free concentration of ZEN in phosphate buffered saline (PBS) after incubation with each of the 27 binders. The amount of ZEN and mycotoxin binder used for incubation is in accordance with the ZEN-binder ratio of 1:20,000, which is based on the maximum guidance level for ZEN in European piglet feed of 0.1 mg/kg [25] and the conventional binder inclusion level of 2 g/kg feed. The individual results of three replicates for the different pHs are presented in a ranked manner (Figure 1) to facilitate comparison between the binders. One-way analysis of variance (ANOVA) for the different binders indicates significant differences in free ZEN concentration (*p* < 0.05). Next, the free ZEN concentration was correlated with the physicochemical characteristics. The correlation matrix of free ZEN concentration and the physicochemical properties is presented in Table 2. 

**Table 1:** Physicochemical characteristics of 27 additives marketed as mycotoxin binders and available in Belgium and The Netherlands. Mean values of triplicate analyses are presented.

Sample Number	XRD Result	HCl	*d*-spacing (10^−10^ m)	CEC (cmol_c_ kg^−1^)	pH	Ca^2+^ (cmol_c_ kg^−1^)	K^+^ (cmol_c_ kg^−1^)	Mg^2+^ (cmol_c_ kg^−1^)	Na^+^ (cmol_c_ kg^−1^)	Swelling (mL)	MF (%)	RH (%)
1	Zeolite	+	9.5	172.9	8.3	16.8	102.4	0.8	24.6	2.1	94.4	4.6
2	Sepiolite, smectite	+	12.4	31.9	7.7	7.3	1.5	9.8	1.3	7.7	96.0	8.7
3	Clinoptilolite	−	10.2	120.3	7.7	8.4	58.7	1.2	10.2	2.5	97.7	4.9
4	Zeolite	−	12.5	413.5	10.3	n.d.	35.4	0.1	363.3	0.0	93.8	7.1
5	Humic substance, quartz	−	26.2	185.9	4.2	7.2	1.5	3.4	19.2	2.5	15.8	10.6
6	Mixed layer montmorillonite, quartz	−	19.1	51.0	7.7	10.0	10.7	3.8	21.8	2.7	78.6	3.4
7	Montmorillonite	++	12.8	82.9	9.8	12.5	2.8	4.0	63.8	43.7	97.1	10.1
8	Montmorillonite	−	15.5	100.5	3.7	19.2	1.8	3.0	0.8	2.2	95.9	13.3
9	Sepiolite, montmorillonite, quartz (t), dolomite (t), albite (t)	+	12.1	39.3	8.2	8.2	0.6	10.2	0.6	7.9	96.3	5.4
10	Montmorillonite, sepiolite, quartz (t), calcite (t)	++	12.4	56.7	8.5	16.9	0.6	8.0	26.9	9.1	96.9	9.1
11	Montmorillonite, quartz (t), calcite (t), feldspars (t)	++	12.6	64.1	9.3	19.6	3.0	6.7	54.3	31.8	98.3	11.9
12	Humic substance, quartz	−	25.9	166.4	4.4	1.3	11.5	0.9	18.4	2.5	6.0	12.4
13	Sepiolite, montmorillonite, calcite (t), quartz (t)	+	12.2	22.1	7.1	17.7	2.2	9.3	4.4	5.9	80.3	6.7
14	Montmorillonite	−	9.2	109.4	5.6	21.7	17.2	1.9	4.2	2.9	92.8	7.2
15	Calcite, dolomite, organic material	++	6.9	12.6	5.7	35.5	19.1	4.2	26.0	7.5	38.9	5.1
16	Thenardite, montmorillonite, quartz, organic material		14.8	7.8	4.1	2.3	26.0	7.0	131.8	4.0	27.3	6.4
17	Montmorillonite	−	12.6	71.8	8.0	9.5	4.0	2.7	49.5	7.6	90.2	9.8
18	Clinoptilolite	−	10.2	176.6	7.4	15.2	44.7	2.0	6.0	2.5	96.3	4.7
19	Quartz, mica, montmorillonite, kaolin	−	14.7	59.7	7.9	18.1	1.9	9.0	0.3	4.3	95.4	7.9
20	Mica, kaolin, quartz, montmorillonite	+	14.7	59.6	7.9	14.4	2.5	8.7	0.6	3.5	97.0	9.0
21	Mixed layered smectite	+	12.4	23.7	9.9	13.3	0.7	19.2	47.7	24.2	97.5	7.5
22	Mica, calcite, smectite	+	15.5	77.9	8.0	33.9	1.8	4.1	0.9	4.3	88.6	11.4
23	Montmorillonite, sepiolite, calcite (t)	++	12.4	46.5	7.9	24.2	1.4	4.7	55.2	8.6	92.7	7.3
24	Montmorillonite, mica, feldspars	−	12.3	7.0	6.2	8.1	12.9	3.3	4.9	3.8	94.8	5.2
25	Calcite, montmorillonite (t)	++	13.1	26.1	6.6	55.8	10.7	2.4	11.6	3.7	97.0	3.0
26	Mixed layered montmorillonite, quartz, feldspars	−	21.5	27.9	7.7	9.3	1.4	2.6	4.9	2.5	98.0	2.0
27	Montmorillonite	−	12.7	111.7	9.5	8.7	1.3	4.0	69.5	5.7	86.8	13.2

**−**, + and ++ indicate minor, moderate and strong reaction in the HCl-effervescence test; n.d., not detectable; CEC, cation exchange capacity; MF, mineral fraction; RH, relative humidity; (t), indicates trace amounts.

**Table 2:** Correlation matrix of the free zearalenone (ZEN) concentration and the physicochemical properties of the 27 mycotoxin binders.

Parameters		Free ZEN concentration pH 2.5	Free ZEN concentration pH 6.5	Free ZEN concentration pH 8.0	Average free ZEN concentration
Free ZEN concentration pH 2.5	R	1	0.887 **	0.874 **	0.948 **
	Sig.	-	0.000	0.000	0.000
Free ZEN concentration pH 6.5	R	0.887 **	1	0.955 **	0.979 **
	Sig.	0.000	-	0.000	0.000
Free ZEN concentration pH 8.0	R	0.874 **	0.955 **	1	0.976 **
	Sig.	0.000	0.000	-	0.000
Average free ZEN concentration	R	0.948 **	0.979 **	0.976 **	1
	Sig.	0.000	0.000	0.000	-
*d*-spacing	R	−0.631 **	−0.632 **	−0.659 **	−0.662 **
	Sig.	0.000	0.000	0.000	0.000
Swelling	R	0.090	0.122	0.182	0.137
	Sig.	0.654	0.545	0.364	0.495
CEC	R	0.319	0.237	0.266	0.282
	Sig.	0.104	0.234	0.179	0.153
pH	R	0.192	0.285	0.357	0.290
	Sig.	0.339	0.149	0.067	0.142
Ca^2+^	R	0.257	0.258	0.256	0.266
	Sig.	0.205	0.204	0.207	0.189
K^+^	R	0.394 *	0.379	0.360	0.389 *
	Sig.	0.042	0.051	0.065	0.045
Mg^2+^	R	−0.399 *	−0.316	−0.227	−0.321
	Sig.	0.039	0.108	0.254	0.102
Na^+^	R	0.302	0.240	0.267	0.278
	Sig.	0.125	0.227	0.178	0.160
RH	R	0.082	−0.006	0.055	0.045
	Sig.	0.684	0.977	0.785	0.824
MF	R	0.421 *	0.419 *	0.525 **	0.472 *
	Sig.	0.029	0.030	0.005	0.013

R: Pearson correlation coefficient; Sig.: significance level; * significant at the 0.05 level (two-tailed); ** significant at the 0.01 level (two-tailed); CEC, cation exchange capacity; pH, acidity of the samples; Ca^2+^, K^+^, Mg^2+^, Na^+^, exchangeable base cations; RH, relative humidity; MF, mineral fraction.

**Figure 2:**
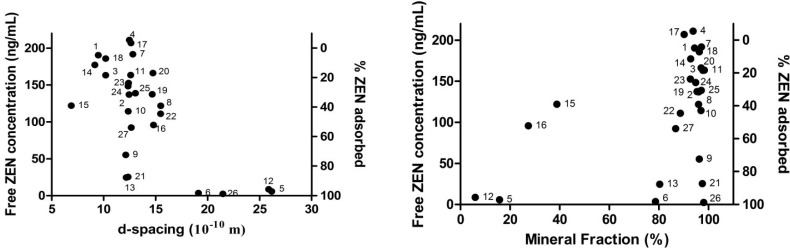
Biplots of the average free concentration of zearalenone (ZEN) with the *d*-spacing (**left**) and the mineral fraction (**right**) of the 27 mycotoxin binders (Numbers 1–27).

A large variability in free ZEN concentration was observed, ranging from 200 ng/mL, which is indicative for no adsorption, to the limit of quantification, which corresponds with 100% adsorption under the given conditions. This is in accordance with previous binding experiments, where a large variability was also observed [18,20]. A significant correlation could be demonstrated between the free ZEN concentration and both the *d*-spacing and mineral fraction (MF). Figure 2 presents the two biplots of these parameters with the free ZEN concentration. In the low pH range (pH 2.5), exchangeable K^+^ and Mg^2+^ were also significantly correlated. The pH may influence the phenolic hydroxyl group of ZEN or the ionization-state of the functional groups of the mycotoxin binders and thereby alter the chemical sorption due to ionic interactions. A low pH can facilitate degradation of the minerals, but this effect is mostly seen over a longer period. Deng *et al.* (2009) described the binding mechanism for aflatoxin B1 (AFB1) to montmorillonite clays, a mechanism involving the exchangeable cations and water [8]. The correlation between the *d*-spacing and the free ZEN concentration suggests a cut off-value between 16 and 19 × 10^−10^ m, as can be seen in the left plot in Figure 2. From this cut off-value, a similar mechanism might apply for ZEN as for AFB1, explaining the low free ZEN concentration in binders expressing a large *d*-spacing. However, some aspects need to be considered: AFB1 has a rather planar structure, which facilitates interlayer adsorption, whereas ZEN has a more spherical molecular geometry. Furthermore, AFB1 is more hydrophilic than ZEN (estimated log P_Aflatoxin B1_ = 1.58 *vs.* estimated log P_ZEN_ = ca. 4.37 [26]). This is important, since the interlayer space is hydrophilic [27]. 

A low free ZEN concentration over the complete pH range was seen with the mixed-layer smectites (Sample Numbers 6, 21 and 26), which was also reported by [20]. The exact mechanism for this remains to be elucidated. XRD and infra-red (IR) spectroscopy of the binding complex can be used to study the role of the *d*-spacing and may unravel the binding mechanism. 

The humic acid-containing binders (Sample Numbers 5, 12 and 13) also presented a low free ZEN concentration. Similar results were observed in three out of five humic substance samples examined by Sabater-Vilar *et al.* [18]. Yeast cell wall-derived products also expressed a low free ZEN concentration, which was also observed by [18] and [19], but not by [20]. A high affinity of organic substances for oxytetracycline and AFB1 was described by [15,28]. The low free ZEN concentration when incubated with organic substances can be explained by the additional binding possibilities that these substances offer. The extra binding possibilities are hydrophobic in nature and comprise van der Waals, π–π and CH-π bonds [14]. Hydrophobic interactions were also suggested for the binding of ZEN to modified Japanese acid clay [24]. In addition, hydrated humic substances are more flexible than the ridged minerals; this flexibility enables a larger interaction surface with the humic substances. These binding possibilities are independent of possible interlayer adsorptions and might be a parallel mechanism for toxin binding, as can be seen in the right plot of Figure 2. The zeolites and sepiolites expressed a rather high free ZEN concentration and are probably not fit for ZEN adsorption. Zearalenone was effectively adsorbed by active carbon, and this was also the case in previously published studies [18,19,20]. 

## 3. Experimental Section 

### 3.1. Mycotoxin Binders, Chemical Products and Reagents

Feed additives marketed as mycotoxin binders (*n* = 27) were collected after a market study to identify the most relevant products. The suppliers include the international companies, Poortershaven, Sanluc, Kemin, Biomin, Alltech, Agrimex, Cenzone tech, Tesgo international, Selko, Clariant, Tolsa, BASF, Miavit, Special Nutrients and American Colloid. Acid-washed sea sand, HCl, CaCl_2_·2H_2_O, NaCl and MgO were supplied by VWR (Leuven, Belgium). Technical ethanol was provided by Fiers (Kuurne, Belgium). The ammonium acetate, boric acid, H_3_PO_4_, Na_2_HPO_4_ and Neβler reagent were provided by Merck (Darmstadt, Germany). Sigma Aldrich (Bornem, Belgium) supplied KCl, MgCl_2_·6H_2_O and glycol. Acros Organics (Geel, Belgium) supplied methyl red, bromocresol green and tert-butyl methyl ether (tBME). Water and acetonitrile (ACN) used for the HPLC analysis were of MS-grade and provided by Fisher Scientific (Wijnegem, Belgium). ZEN and ^13^C_18_-ZEN were purchased from Fermentek (Jerusalem, Israel) and Romerlabs (Tulln, Austria), respectively.

### 3.2. CEC and Exchangeable Base Cations

A glass burette with a porous bottom was filled with, respectively, 10 g of acid-washed sand, 25 g of acid-washed sand that was thoroughly shaken on a horizontal shaker for 30 min with 0.5 g of binder and 5 g of acid-washed sand to avoid splattering. After 20 min of equilibration, 150 mL of technical ethanol was percolated over the burette for two hours. Next, 150 mL of a 1 mol/L aqueous ammonium acetate solution was percolated in the same manner for a total time of 4 h. The ammonium acetate percolate was analyzed with inductive coupled plasma-atomic emission spectrometry (ICP-AES), as described by Burt *et al.* [7]. The device used was an IRIS Interpid II XSP (Thermofisher, Waltham, UK). The characteristic wavelengths used were 317.9 nm for Ca^2+^, 766.4 nm for K^+^, 285.2 nm for Mg^2+^ and 589.5 nm for Na^+^. 

After the ammonium acetate percolation, the column was rinsed with 150 mL of technical ethanol to remove ammonium that was not adsorbed by the sample. This washing step was performed over a period of 2 h, respecting 20 min of equilibration. The percolate was tested for the presence of ammonia with the Neβler reagent. In case the test was positive, an extra 100 mL of ethanol was used to remove all ammonia. Next, 500 mL of KCl 1 mol/L were percolated over 4 h, again respecting 20 min of equilibration. Fifty milliliters of the KCl percolate were transferred to a Buchi-tube (Buchi labortechnik AG, Flawill, Switzerland), together with about 5 g of MgO. Ammonia was captured in a boric acid-containing solution (20 mL, 0.3 M). The boric acid solution was supplemented with indicators methyl red and bromocresol green. The formed tetrahydroxyborate was titrated back to boric acid with 0.01 mol/L of HCl, and the titration was considered complete when the red color reappeared. The reactions involved and formulas to calculate the CEC value are presented below:

(1) $${NH}_{4}^{+}\overset{T↑;MgO}{⟶}{NH}_{3}\left(↑\right)$$

(2) $${NH}_{3}+B{\left(OH\right)}_{3}+H_{2}O\to {NH}_{4}^{+}+{B(OH)}_{4}^{-}$$

(3) $${B(OH)}_{4}^{-}+{H_{3}O}^{+}⇋B{\left(OH\right)}_{3}+2H_{2}O$$

(4) $${CEC}_{pH7}=\frac{\left(V_{2}-V_{0}\right)\times T\times V\times 100}{V_{1}\times G}$$

with (V_2_−V_0_) representing the volume of HCl used, T the titer of HCl (=0.01 mol/L), V the volume of KCl percolate (=500 mL), V_1_ the volume of KCl percolate sample (=50 mL) and G the mass of the binder (=0.5 g). A KCl solution was used as a blank sample for the titration; a pure sand sample was included for the percolation [7,29]. 

### 3.3. Other Characterization Tests

To measure the acidity of the samples, a 1:10 binder:water suspension was shaken for 2 h and was left to sediment for another 2 h under closed lid. The pH of the supernatant was measured using a glass-calomel electrode (Inolab WTW, Weilheim, Germany).

The presence of carbonates in the samples was tested with a HCl effervescence test: a small amount of binder was mixed with a few droplets of concentrated HCl on a glass dish. The reaction in the first 10 seconds was monitored and scored as follows: −, no reaction; +, moderate reaction; ++, strong reaction.

To determine the relative humidity and the mineral fraction, 10 g of binder were dried in an oven (Memmert, Swabach, Germany) at 110 °C overnight. The sample was weighed before and after drying, and the moisture content was calculated based on the weight reduction. The mineral fraction was assessed by the dry combustion method by heating in a Muffle^®^ furnace (Nabertherm, Lilienthal, Germany) to 400 °C for 16 h and then cooled in a desiccator [7].

The swelling volume was assessed by using an adaptation of the coefficient of linear extensibility (COLE) [7,21]. An aliquot of the binder (2.5 mL tapped bulk volume) was mixed with 15 or 50 mL of water, depending on the extent of swelling. The mixture was thoroughly vortexed (15 s) in the volumetric tube, incubated (4 h) and centrifuged (1070× *g*, 10 min, 4 °C) before measuring the volume of the sediment.

XRD patterns, including *d*-spacing, were obtained with a Philips X'PERT SYSTEM (Phillips, Eindhoven, The Netherlands), the diffractometer (type: PW 3710) was equipped with a copper tube anode, a secondary graphite beam monochromator, a proportional xenon filled detector and a 35-position multiple sample changer. The incident beam was automatically aligned, and the irradiated wavelength was 12 mm. The secondary beam side surpassed a 0.1-mm receiving slit, a Soller slit and a 1° anti-scanner slit. The tube was operated at 40 kV and 30 mA. XRD data were collected in a theta, 2-theta geometry from 3.00' onwards at a step of 0.020° 2-theta and a counting time of 1 s per step. XRD patterns of powder samples, oriented samples and glycol-saturated oriented powder samples were recorded.

### 3.4. Zearalenone Adsorption Screening

A saline solution was made by adding 24.0 g of NaCl, 0.3 g of MgCl_2_·6H_2_O, 0.6 g of KCl and 0.4 g of CaCl_2_·2H_2_O to 3L HPLC-grade water. Next, a phosphate buffer system was added to 1 L of saline solution to obtain phosphate buffered saline (PBS). The buffer system consisted of H_3_PO_4_ and KH_2_PO_4_ for the acidic (pH 2.5) buffer and of KH_2_PO_4_ and Na_2_HPO_4_ for the buffers of pH 6.5 and 8.0. Total buffer concentration was calculated with the Henderson–Hasselbalch equation and the constraint to obtain a total osmolarity of 9.6 mmol/L in each buffer. The pH was measured and adjusted with H_3_PO_4_ or Na_2_HPO_4_ to obtain buffers of pH 2.5, 6.5 or 8.0. A 60-mL flask was filled with 20 mg of each of the binders and 5 mL of PBS; this was done for each pH, in triplicate. ZEN was added to a final concentration of 200 ng/mL. The flask was then shaken for 4 h at 37 °C in an incubator (New Brunswick Scientific, Rotselaar, Belgium). Next, samples were centrifuged (10 min, 1070× *g*, 25 °C), and 2 mL of the supernatant were transferred to a test tube. Next, 25 µL of the internal standard (IS, ^13^C_18_-ZEN, 1 µg/mL) were added and vortexed, followed by 4 mL of tBME. The tube was swirled on a roller bench (Stuart Scientific, Surrey, UK) for 20 min and centrifuged (10 min, 2,851× *g*, 4 °C). The supernatant was evaporated to dryness under a gentle nitrogen stream (40 ± 5 °C). The dry residue was reconstituted in 200 µL of ACN and transferred to a glass vial for LC-MS/MS analysis. 

The HPLC system consisted of a Waters 2690 pump and autosampler system with a Zorbax Eclipse C-18 HPLC column (3 mm × 100 mm; i.d. 3.5 µm) and a pre-column of the same type (Agilent, Diegem, Belgium). The injection volume was 10 µL. The mobile phases were ACN (A) and HPLC-grade water supplemented with 0.3% ammonia (B). The gradient elution program was as follows: 0–0.5 min, 50% A/50% B; 0.5–1 min, linear gradient to 70% A/30% B; 1–4.5 min, 70% A/30% B; 4.5–5.5 min, linear gradient to 50% A/50% B; 5.5–8 min, 50% A/50% B. The flow rate was set at 0.6 mL/min. The MS/MS detection system was a Micromass Quattro Ultima (Micromass, Manchester, UK) operated in the ESI-negative mode. The *m*/*z* transitions for quantification were 335 > 140 (^13^C_18_-ZEN) and 317 > 131 (ZEN). The capillary and cone voltages were −3.47 kV and 60 V, respectively, and source temperature was set at 120 °C and desolvation temperature at 200 °C. The cone gas flow and desolvation gas flow were set at 848 L/h and 60 L/h, and the optimized collision energy was 30 eV.

The analytical method was validated for the three pHs independently according to European guidelines (2002/657/EC, 2002) and was adapted from the method by De Baere *et al.* (2012) [30]. The validation included evaluation of linearity, within- and between-run accuracy and precision, limit of detection (LOD), limit of quantification (LOQ), specificity and carry-over. The correlation coefficients (*r*) and goodness-of-fit coefficients (*g*) of the 7-point calibration curves were calculated and fell within the limits of specification, ≥0.99 and ≤10%, respectively. For the precision, the relative standard deviation (RSD, %) fell within 2/3 of the values calculated according to the Horwitz equation, RSD_max_ = 2^(1−0.5logConc)^ × 2/3, for within-run precision, with a minimum of 10%, and within the values calculated according to the Horwitz equation for between-run precision, RSD_max_ = 2^(1−0.5logConc)^. The LOQ was determined by analyzing six samples spiked at 3.13 ng/mL, on the same day. Detection limits for pH 2.5, 6.5 and 8.0 were respectively 0.70, 1.07 and 0.66 ng/mL. 

### 3.5. Statistical Analysis

Analysis of variance (ANOVA) was used to compare the free concentration of ZEN for the different binders. The free ZEN concentration was correlated with the continuous explanatory variables. *p*-values below 0.05 were considered statistically significant. All analyses were conducted using SPSS 22 (IBM, Chicago, IL, USA), and graphs were obtained with GraphPad Prism^®^ version 5 (La Jolla, CA, USA).

## 4. Conclusions

Twenty-seven frequently-used feed additives and marketed as mycotoxin binders were characterized. A single concentration *in vitro* adsorption screening of ZEN was executed in three different PBS-buffers (pH 2.5, 6.5 and 8.0). A significant correlation between free ZEN concentration and both the *d*-spacing and mineral fraction could be demonstrated. In the low pH range (pH 2.5), an additional correlation between the exchangeable K^+^ and Mg^2+^ could be demonstrated. Humic acid-containing binders and mixed-layered smectite-containing binders achieved the lowest free ZEN concentration.
